# The Food, Feelings, and Family Study: comparison of the efficacy of traditional methods, social media, and broadcast email to recruit pregnant women to an observational, longitudinal nutrition study

**DOI:** 10.1186/s12884-021-03680-1

**Published:** 2021-03-12

**Authors:** Rebecca Smith, Crystal Alvarez, Sylvia Crixell, Michelle A. Lane

**Affiliations:** Nutrition and Foods Program, School of Family and Consumer Sciences, Texas State University, 601 University Dr., San Marcos, TX 78666 USA

**Keywords:** Recruitment, Pregnant, Depression, Nutrition, Social media

## Abstract

**Background:**

It is well known that recruitment is a challenging aspect of any study involving human subjects. This challenge is exacerbated when the population sought is reticent to participate in research as is the case with pregnant women and individuals with depression. This paper compares recruitment methods used for the Food, Feelings, and Family Study, an observational, longitudinal pilot study concerning how diet and bisphenol A exposure affect maternal mood and cognitive function during and after pregnancy.

**Methods:**

Pregnant women were recruited to this study over a period of 15 months using traditional methods, social media including paid and unpaid posts, and emails broadcast to the university community. Contingency analysis using the Pearson’s Chi-square test was used to determine if recruitment method was associated with likelihood of participation. T-tests were used to analyze Facebook advertisement success. ANOVAs and Fisher exact tests were used to determine if recruitment method was related to continuous and categorical demographics, respectively.

**Results:**

Social media resulted in the largest number of recruits, followed by traditional methods and broadcast email. Women recruited through social media were less likely to participate. In contrast, use of broadcast email resulted in a smaller pool of recruits but these recruits were more likely to be eligible for and complete the study. Most women recruited via social media were the result of unpaid posts to the study’s Facebook page. Paid posts lasting at least 4 days were the most successful. Recruitment method was not associated with participant demographics.

**Conclusions:**

Social media has the potential to recruit a large pool of potential subjects; however, when studies require a large time investment such as the case here, women recruited through social media are less likely to participate and complete the study than women recruited through other means.

**Trial registration:**

N/A. This study does not describe a health care intervention.

**Supplementary Information:**

The online version contains supplementary material available at 10.1186/s12884-021-03680-1.

## Background

Pregnancy, a critical period in the life of many women, is often associated with mood disorders [1, 2]. There are gaps in the literature, in particular concerning the relationship between a woman’s diet and her mood and cognitive function during and following pregnancy. The gaps may exist because few pregnant women participate in research trials [3–7] and they have historically been classified as vulnerable human subjects because study participation could adversely affect pregnancy outcomes [4]. More recently, this categorization of pregnant women as a vulnerable population has been challenged, and viewed as an affront to women’s autonomy [8, 9]. When asked why they do not chose to participate in scientific research, pregnant women report that they: (i) lack interest in [6, 10] or distrust scientific research [9], (ii) feel as though preparing for the arrival of the baby leaves them with no time to participate [5, 6, 9], (iii) lack transportation to a study site [11, 12], (iv) experience disapproval from family and friends [5, 13], and (v) may be experiencing pregnancy-related health problems [5].

Compounding the problem, the symptoms of pregnancy-related depression, estimated to be present in up to 20% of women [1, 2], interfere with participation in research studies. Specifically, low motivation, lack of self-confidence, and perceived stigma are relevant barriers (systematically reviewed in [14]). In addition, the comorbidity of attention deficit disorders and depression [15] and the reported lack of attention due to pregnancy itself [16] may prevent the completion of tasks, potentially reducing the likelihood that pregnant women and particularly those with pregnancy-related depression will participate in long-term research activities. This adds to the difficulty inherent in recruiting for longitudinal studies as long-term time commitments deter participation [17, 18]. Nutrition-related studies also present unique barriers to participation [19, 20]. In particular, the collection of dietary intake data requires a substantial time commitment from the participant [21, 22]. In short, collectively, pregnancy, depression, longitudinal design, and issues surrounding dietary intake studies converge to make recruitment challenging, exhaust researcher efforts, delay the completion of studies, and impact methodological quality and study validity (reviewed in [12]). Of note, depending on the study design and participation criteria, 19 to 30% of clinical trials fail to meet enrollment targets [23, 24]. In light of these problems, it is imperative to understand which methods are the most effective to recruit and enroll challenging populations, such as pregnant women experiencing depression.

There are several approaches to recruit study participants, including traditional methods, social media, and email. Traditional recruitment methods include, but are not limited to, newspaper ads, posters, flyers, and word-of-mouth. Electronic recruitment via social media and email has the potential to have a broader reach than traditional methods. To wit, approximately 96 and 77% of US adults ages 18–29 years have a smartphone and home broadband, respectively [25, 26]. Further, social media recruitment methods typically involve services such as Facebook and Instagram, which are accessed by approximately 90% of adults in this age group [27].

The Food, Feelings, and Family (FFF) Study, launched in April 2018, was a pilot study aimed to recruit low income women experiencing pregnancy-related depression in their third trimester of pregnancy. Specifically, this longitudinal study was designed to investigate the effects of the antenatal diet and bisphenol A (BPA) exposure on ante- and postnatal depression and cognition. Because we encountered numerous barriers when attempting to recruit this understudied population, the purpose of this report is to share lessons learned to facilitate future research.

## Methods

### Overview of the food, feelings, and family study

**Fig. 1:**
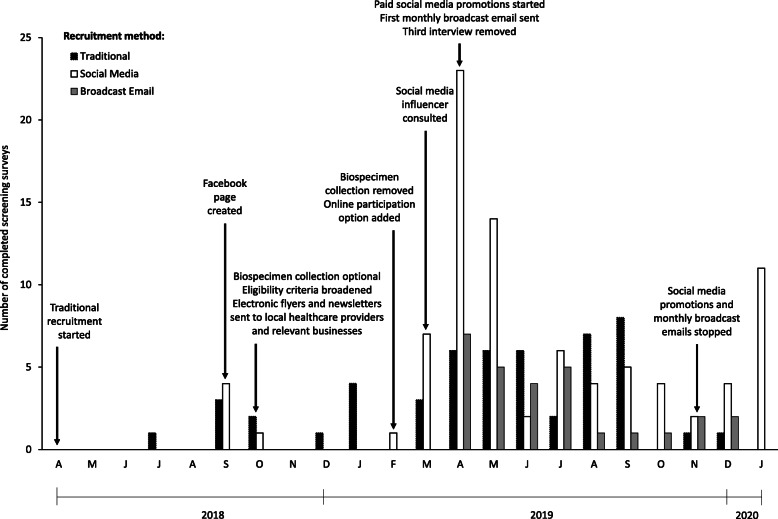
Study timeline versus number of screening surveys completed. Black, white, and gray bars indicate the number of participants that completed the screening survey as a result of traditional recruitment practices, social media, and broadcast email, respectively, as described in the Methods section. Social media refers to both Facebook and Instagram. Alterations to the study protocol to increase participation are indicated by arrows. Letters on the x-axis indicate the first letter of each month of the year, starting with April represented by the letter A

The FFF Study recruited participants from April, 2018 to January 2020 (Fig. 1). In the original study design, eligible participants were to meet face-to-face with researchers on three occasions: during the third trimester of pregnancy (antenatal interview), 2–4 weeks postpartum, and 12 weeks postpartum. During these meetings, participants were to perform various tasks such as providing blood and urine samples and completing demographic, health, depression, and anxiety surveys, a diet history questionnaire, and a cognitive test. For the purposes of this report, “recruitment” refers to the number of individuals who completed the screening survey while “participation” reflects completion of the entire study.

Initially, the study aimed to recruit 144 low-income pregnant women, half of which were experiencing pregnancy-related depression as assessed by the Edinburgh Postpartum Depression Scale (EPDS). The EPDS is a ten-question self-rating scale developed to assess depressive symptoms in the postnatal period [28]. This scale can also be used to identify depression in the antenatal period [29–31]. Successful recruitment would yield a final sample of 100 based on an anticipated attrition rate of approximately 30% [32]. After 6 months of unsuccessful recruitment, however, we eliminated the requirements for biological specimen collection, EPDS scores reflective of pregnancy-related depression, as well as income restriction, in hopes of attracting more potential participants (Fig. 1). In addition, 4 months later, when the Psychology Experiment Building Language (PEBL), the cognitive testing software used for this study, became available online for both Windows and Macintosh operating systems, we were able to eliminate the need for face-to-face interviews and thereby engage participants online, allowing us to reach a national audience. Finally, 1 year after recruitment began, the third interview was eliminated to further motivate participation and retention.

The final inclusion criteria were: (1) ages 18–35 years, (2) singleton pregnancies and (3) 28–37 week’s gestation at the antenatal interview. The exclusion criteria were: (1) use of brain training games (e.g. Lumosity), (2) multifetal pregnancies, (3) diagnosis of mental illness other than perinatal and major depression, and (4) lack of internet access. Women that were interested in our study were invited to complete the eligibility screening survey, hosted in Qualtrics, which included questions about demographics, medical history, use of brain training games, and the EPDS. Brain training games mimic the cognitive tests used for this study and have potential to confound the interpretation of cognitive data [33–35].

Participation in the FFF Study required two interviews and the completion of several assessments. Women were invited to participate face-to-face or online. Face-to-face participants were provided with a password protected computer dedicated to the project to complete the interviews. Participants who opted to participate online were required to have a webcam, microphone, and computer. The antenatal interview at 28–37 weeks gestation lasted approximately 1 h during which time participants completed demographic, mood [28, 36, 37] and sleep surveys [38], the Diet History Questionnaire III [39], cognitive failures questionnaires [40], and cognitive tests [41–43]. Between the ante- and postnatal interviews, participants were asked to submit the BPA Exposure Assessment Module [44] and complete three 24-h dietary recalls via the Automated Self-Administered 24-Hour Dietary Assessment Tool [45]. The postnatal interview, held 2–4 weeks following birth, was approximately 30–45 min in length, and required participants to complete surveys regarding mood [28, 36, 37], sleep [38], and cognitive failures [40], and undergo cognitive testing [41–43].

Participants were incentivized with $100 in Amazon gift cards distributed after completion of various tasks related to the study. In addition, following the antenatal interview participants received “belly butter.” Further, following completion of the postnatal interview, participants were given a baby bib with the FFF Study logo and a personalized dietary analysis. These gifts were mailed to online participants. All study aspects were approved by the Institutional Review Board of Texas State University, protocol #4897. Written informed consent was obtained from all subjects.

### Traditional recruitment procedures

**Fig. 2:**
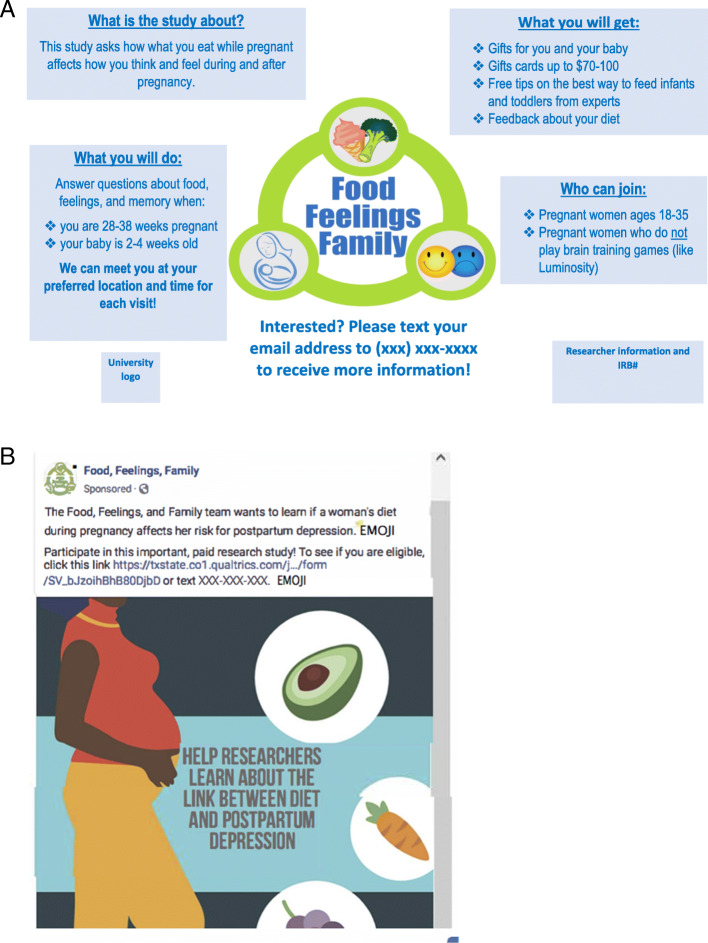
Sample recruitment flyer and example of a Facebook paid promotion. The recruitment flyer (**a**) contains the Food, Feelings, and Family logo and contact information (university logo is not shown due to copyright) and information regarding the study’s objective, participation expectations, incentives, eligibility criteria, and investigator information. The Facebook promotion (**b**) displays the study’s logo and objective, a link to the screening survey, contact phone number, and text indicating where emojis depicting fruits and vegetables were located. All images other than the university logo (**a**) and the emojis (**b**) were original or in the public domain

From the onset of the FFF Study we employed a comprehensive approach to traditional recruitment throughout the Central Texas area, spanning from San Antonio to Pflugerville, TX and covering four counties. For example, flyers advertising the FFF Study (Fig. 2a), including the logo, study purpose, basic eligibility requirements, incentives offered, and contact information were distributed to healthcare facilities and businesses catering to pregnant women or parents of young children, such as obstetrician offices, birthing centers, public health clinics, child care centers, childrens clothing stores, and libraries. Flyers were also posted across the university campus and in community centers and churches. In addition, booths were hosted at medical centers, low income housing facilities, and food banks. Also, free classes related to infant nutrition and feeding healthful complementary foods to young children were offered on the university campus and at various facilities. Finally, researchers visited approximately 50 potential recruitment sites within a 50-mile radius of their campus such as obstetrician-gynecologists, midwives, doulas, and a large hospital network in central Texas to establish relationships with these stakeholders. Thereafter, these stakeholders were visited at least once each month and suppled with additional recruitment flyers (Fig. 2a) and monthly newsletters (Additional Files 1 and 2) either on paper or via email for distribution to potential participants as per their preference. Incentives were not offered to these stakeholders. The newsletters included topics likely to be of interest to pregnant women, such as how to find credible sources of information related to medicine and science (Additional File 1), child nutrition (Additional File 2), lactation, and pregnancy massage. The hospital network presented information regarding the FFF Study at a meeting of their obstetrician-gynecologists and distributed the flyers and monthly newsletters to obstetrician-gynecologists within their network for use in participant referrals. Despite these efforts, few women completed the screening survey: therefore, we decided to expand our efforts to include recruitment via social media and broadcast emails.

### Social media recruitment procedures

A Facebook page and Instagram account entitled “Food, Feelings, Family” and displaying the study logo, a link to the study’s webpage, a description of the research team, and contact information were created early in the study (Fig. 1). A social media influencer was consulted to provide insight regarding how to build a Facebook and Instagram following. Based on her recommendations, the researcher organizing the FFF page asked to join Facebook groups likely to have expectant mothers or women experiencing perinatal depression as members (Additional File 3). Terms used to identify potential groups included: pregnancy, pregnant, mother, mom, mommy, perinatal, depression, and anxiety. For private groups, researchers contacted the group administrator and asked permission to join. In addition, health care providers that were located in Central Texas were contacted via Facebook and asked to share the FFF Study recruitment flyer on their pages. Instagram was used to follow the accounts of healthcare professionals and pregnancy related entities. In return, these accounts could then follow the FFF Study Instagram and share our information to their feed.

Strategies aimed at leveraging the Facebook page to recruit participants included (i) sharing of posts to FFF Study researchers’ personal Facebook pages, (ii) shared posts to Facebook groups, and (iii) paid Facebook/Instagram promotions. Utilizing graphic design platforms, posts related to the study in general or postpartum depression in particular were created. There was a total of 73 posts published on the FFF Facebook page. The 59 unpaid posts provided general information about our study, perinatal depression, the FFF research team, opportunities to attend free infant feeding classes, and date-specific salutations (e.g. “The FFF team wishes you a Happy 4th of July!”). The 14 paid posts included (1) a stock image or icon (e.g. pregnant mother, mom and baby, food icons), (2) an engaging quote or brief text (e.g. “Share with any mommy-to-be!”), (3) information about our study, and (4) a link to our screening survey (Fig. 2b). The paid Facebook posts were promoted from April to November 2019. The Facebook Ads manager allows the user to set a post objective (e.g. increase page and post awareness) and target audience (e.g. gender, age, and interests). This allowed paid posts to be targeted to Facebook users that were female, ages 18–50, and residing within the US. Posts were promoted for 1–7 days, at a cost of approximately $5 per day. Paid posts had a set budget and Facebook performed automatic bidding to achieve the greatest number of clicks for the lowest cost. All paid promotions were shared to Facebook mobile, Facebook desktop, and Instagram news feeds. Facebook or Instagram users that viewed our paid promotions could opt to visit our study page, click the link to our screening survey, like, comment, or share our posts.

### Broadcast email recruitment

In addition to social media recruitment, a broadcast email was sent to approximately 4000 university faculty and staff on a monthly basis between April and November, 2019 (Fig. 1). Each email included: (i) the study’s goals, (ii) inclusion criteria, (iii) incentives, (iv) ways to participate (e.g. in person or online), (v) a brief overview of what participation would entail, and (vi) researchers’ contact information. Recipients of the email were encouraged to share study information with potential participants.

### Data collection and statistical analyses

All recruits indicated how they learned about the FFF Study in the online screening survey. Contingency tables using the Pearson’s Chi-squared test were used to determine if recruitment method was associated with likelihood to complete the study. A *P*-value < 0.05 indicated significance for the overall comparison (e.g. recruitment method x eligibility). Post-hoc tests for significant comparisons were conducted by calculating Z-scores (the standardized adjusted residual) for each combination of recruitment method and the categorical variable of interest. Z-scores were converted to estimated *P*-values by squaring each Z-score then calculating the right-tailed probability using the Chi-square distribution with one degree of freedom. The resulting *P*-value was compared to the adjusted significance value identified using the Bonferroni correction to reduce the likelihood of Type I error resulting from multiple comparisons (*P* = 0.008).

The Facebook Ads Manager provided data for each paid advertisement, including the number of days the promotion ran, post impressions, post engagement, and cost per click. Post impressions quantify the number of times our post was delivered to a user’s Facebook newsfeed. Post engagement reflects the number of times an action such as a like, share, or comment was made on each post. The insights tab on the FFF Facebook page provided data regarding the total number of posts on the FFF Facebook feed as well as paid reach. Paid reach specifies the number of Facebook users that had a paid post appear on their screens. With the exception of cost per click, the effects of paid post duration and subject on these metrics were compared using a Welch’s t-test for unequal variances. Differences in cost per click were determined via Student’s t-test. These data are displayed as mean ± SD in the text.

Demographic information was self-reported by participants during the antenatal interview. An ANOVA was used to determine if age, income, gestational age, and EPDS score differed by recruitment method. Due to the relatively small sample size and expected cell counts, Fisher Exact Tests were used to determine if the recruitment method was associated with ethnicity, education, poverty, and pregnancy number. All analyses were performed using IBM SPSS Statistics for Macintosh, Version 24.

## Results

### Recruitment and enrollment

The traditional recruitment method utilized during the first 11 months of the FFF Study yielded only 14 completed online screening surveys (Fig. 1). Also, during this time, 13 individuals completed screening surveys due to visiting our Facebook page or Instagram feed. Responses to our screening survey increased dramatically in April 2019 following the implementation of paid social media promotions on Facebook and broadcasting emails to the university faculty and staff.

**Fig. 3:**
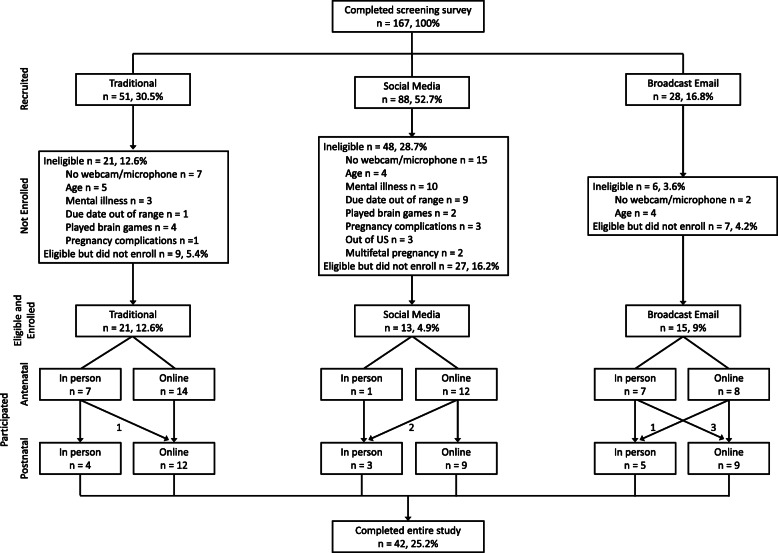
Flowchart of participation by recruitment methods from screening to study completion. The term “Social Media” includes both Facebook and Instagram. Diagonal arrows indicate the number of participants that changed from in person to online interviews and vice versa. Percentages represent the percent of total recruits and may not add up to 100 due to rounding

Altogether, 172 individuals began the FFF screening survey; of these, 167 completed it, and were considered “recruited” (Fig. 3). The majority of individuals were recruited via social media (*n* = 88), including Facebook and Instagram, followed by traditional methods (*n* = 51), and broadcast email (*n* = 28). Of these, 12 women recruited via social media, 16 via traditional methods, and 14 via broadcast email completed the study. Interestingly, the likelihood of a recruit to complete the study differed by recruitment method (*P* = 0.0003)*.* Specifically, fewer women recruited via social media (*n* = 12; *P* = 0.0003) and more women recruited via broadcast email (*n* = 14; *P* = 0.0009) than expected completed the study. There was also a difference in eligibility between recruitment methods (*P* = 0.007); women recruited via email (*P* = 0.008) were more likely to be eligible for the study. Forty-one, 54.5, and 21.4% of women recruited using traditional methods, social media, and broadcast email were not eligible to participate in the study, respectively. Reasons for ineligibility are shown in Fig. 3. The primary reason was lack of access to a computer with a microphone or a webcam necessary for online participation (*n* = 24). When only eligible recruits were considered, the likelihood of completing the study differed by recruitment method (*P* = 0.023). In summary, while social media resulted in the largest number of completed screening surveys, broadcast email, while resulting in a lower number of completed screening surveys overall, produced a pool of recruits that were more likely to be eligible for the study.

### Facebook metrics

The FFF Facebook page, active for 58 weeks, received 225 views, 102 likes, and 111 followers. As mentioned above, prior to using paid posts to promote our study on Facebook, 13 women that completed the screening survey indicated they heard about the FFF Study on social media (Fig. 1). After we increased the visibility of the FFF Facebook page in March 2019 by joining or contacting other Facebook pages (Additional File 3), 27 individuals completed the screening survey. Our first paid Facebook promotion on April 30, 2019 corresponded to Maternal Mental Health Awareness Week [46]. This promotion was followed by 13 additional paid and promoted Facebook posts over a period of 6 months. Thirty-nine women completed screening surveys as a result of paid Facebook promotions between April 30 and November 17, 2019 (Fig. 1). The total cost of these promotions was approximately $265 (Table 1). Post success was measured by greater paid reach, impressions, and engagement, and lower cost per click. The success of posts was related to post duration but not subject (i.e. FFF Study or postpartum symptoms or statistics). Specifically, promotion of a post for at least 4 days, as opposed to 3 or fewer days, resulted in greater paid reach (2501 ± 1300 vs 239 ± 79, *P* = 0.001), a higher number of impressions (2679 ± 1354 vs 242 ± 81, *P* = 0.001), and more engagement (366 ± 194 vs 53.2 ± 20, *P* = 0.001). Cost per click did not differ by post duration (*P* = 0.143). Interestingly, 15 of the women that completed the screening survey indicated they found out about the FFF Study on social media after termination of paid Facebook promotions (Fig. 1).

**Table 1:** Paid Facebook post success metrics for the FFF Study

Post Number	Days Promoted	Subject^a^	Paid Reach	Impressions	Engagement	Total Cost	Cost Per Click
1	1	FFF	288	307	53	$5.00	$0.16
2	1	PPD	113	116	24	$4.93	$2.47
3	1	PPD	302	302	67	$4.98	$0.31
4	1	PPD	210	208	46	$4.99	$4.99
5	3	PPD	280	277	76	$10.00	$5.00
6	5	PPD	3154	3348	695	$20.00	$1.25
7	5	FFF	2103	2190	271	$20.00	$0.39
8	5	FFF	3802	4076	285	$25.00	$1.19
9	5	FFF	1716	1842	286	$20.00	$1.18
10	7	FFF	1774	1904	256	$30.00	$0.32
11	7	FFF	1384	1402	263	$30.00	$4.29
12	4	FFF	1833	1953	262	$19.84	$0.16
13	6	PPD	5227	5501	719	$39.97	$0.68
14	5	FFF	1516	1899	288	$30.00	$0.21

^a.^ Subject refers to post content related to the Food, Feelings, and Family (FFF) Study in general or postpartum depression (PPD) symptoms or statistics

### Recruit location and participant demographics

**Fig. 4:**
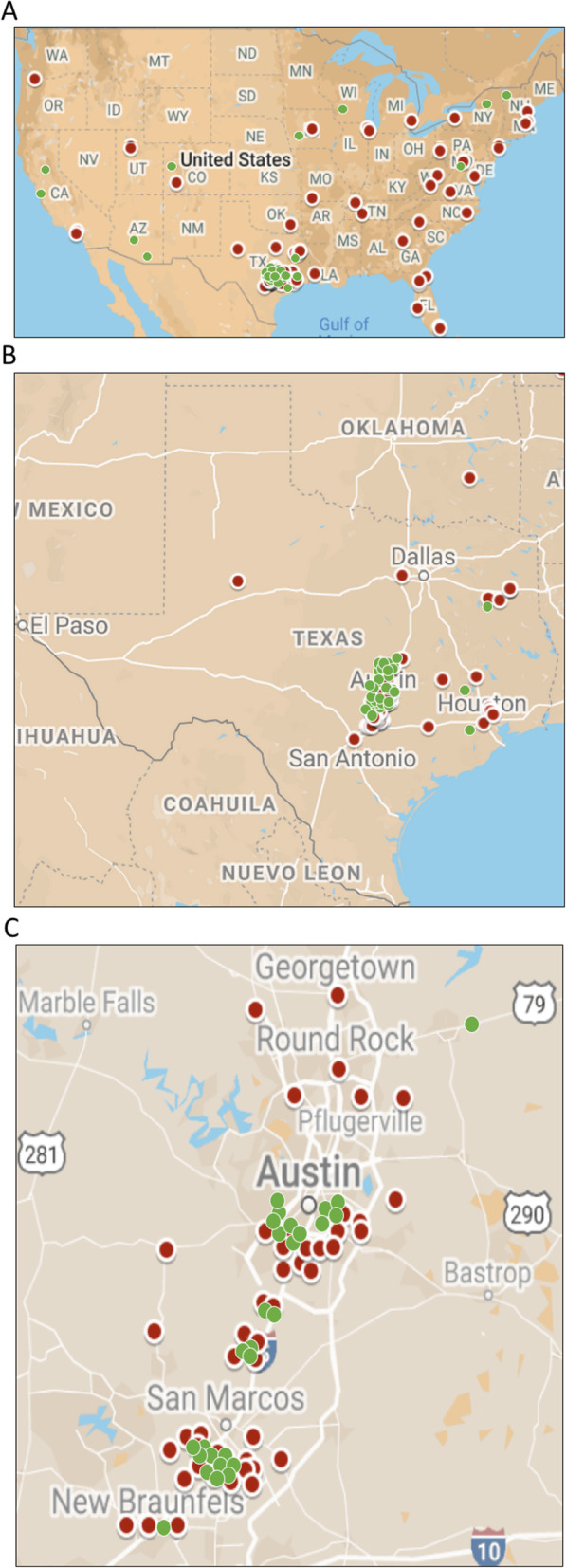
Geographical distribution of recruits and participants in the Food, Feelings, and Family Study. Google maps was used to indicate locations across (**a**) the United States, (**b**) Texas, and (**c**) Central Texas. Red circles indicate women that completed the screening survey (recruits). Green circles display the location of study participants. In (C) blue triangles indicate the location of cities and towns in Central Texas. The three recruits that completed the screening survey and lived outside the US are not included. The map of the United States was modified from the original [47]. The maps of Texas and Central Texas were derived from [48]. These maps are available under the GNU General Public License

Screening survey respondents included women across the US residing in 28 out of the 50 states (Fig. 4a). Despite recruiting nationwide, most respondents lived in TX (*n* = 112; Fig. 4b). In fact, the majority respondents were from the Central Texas area (Fig. 4c), including the cities of Austin (*n* = 37), San Marcos (*n* = 29), Kyle (*n* = 7), Buda (*n* = 5), and New Braunfels (*n* = 4). With respect to participants, only 13 lived outside of the Central Texas area.

Recruitment method was not related to participant age, income, gestational age, or EPDS score at screening (Table 2). In addition, the EPDS scores were not reflective of pregnancy-related depression. There were no differences in ethnicity, education, poverty, or pregnancy number due to recruitment method. All participants recruited via the broadcast email had completed college or some graduate/professional school with income above the poverty level. Women recruited through traditional methods and broadcast email were mostly primiparous.

**Table 2:** Demographic characteristics of participants that completed the FFF Study by recruitment method

Characteristic	Traditional Methods (***n*** = 16)	Social Media (***n*** = 12)	Broadcast Email (***n =*** 14***)***	***P-***value
Age (years)	29.8 ± 5.1^a^	30.1 ± 2.9^a^	30.5 ± 3.0^a^	0.872
Annual household income ($)	76,474 ± 60,317	76,067 ± 43,922	113,621 ± 64,490	0.156
Gestational age (weeks)^b^	34.1 ± 1.7	33.6 ± 1.8	33.9 ± 1.9	0.783
EPDS^c^ score at screening	6.3 ± 3.8	7.0 ± 3.0	6.3 ± 3.0	0.814
Ethnicity				0.318
White	7 (43.8)^d, e^	5 (41.7)^d^	10 (71.4)^d^	
Hispanic or Latinx	8 (50.0)	5 (41.7)	2 (14.3)	
Black or African American	1 (6.3)	1 (0.8)	1 (7.1)	
Other	0	1 (0.8)	1 (7.1)	
Education				0.319
12th grade	4 (25)	2 (16.7)	0	
College	7 (43.8)	6 (50)	6 (42.9)	
Graduate/Professional school	5 (31.3)	4 (33.3)	8 (57.1)	
Pregnancy				0.773
First	9 (56.3)	5 (41.7)	8 (57.1)	
Second	6 (37.5)	7 (58.3)	5 (35.7)	
Third or greater	1 (6.3)	0	1 (7.1)	

^a^ Mean ± standard deviation

^b^ At antenatal interview

^c.^ Edinburgh postpartum depression scale (EPDS)

^d.^ n (%)

^e.^ Percentages may not add up to 100 due to rounding

## Discussion

The FFF was an ambitious pilot study, aimed at addressing important gaps in the literature related to diet, BPA exposure, mood, and cognition during and after pregnancy. Importantly, this study is unique because we focus on how the mother’s diet affects her mental state, whereas most studies concern how maternal diet impacts child-related outcomes (e.g. [49–51]). Despite extensive efforts over a time span of almost 2 years, we were unable to reach our goal of recruiting 144 participants. In the current report, we compare three recruitment strategies: traditional, social media, and broadcast email in an attempt to determine which was the most successful at reaching this elusive population. We found that while social media posts generated the greatest number of completed screening surveys, these women were less likely to enroll and participate in the study.

Pregnant women report that they do not participate in research studies because they often lack interest in or distrust scientific research [6, 9, 10], experience disapproval from friends and family [3, 5], and feel as though they do not have time to participate [5, 9]. On the other hand, pregnant women indicate they are motivated to participate when they receive personal benefits, including the potential for improved pregnancy outcome [52, 53], health education [54], and improvements to their own health [10, 52]. Altruistic reasons, such as helping future patients [53] and contributing to knowledge, also spur participation in research studies [9, 10, 52, 54–56]. For example, Daniels et al. [55] found that 93% of pregnant women were motivated to participate in a study because they would contribute to science. Our social media posts included text encouraging women to “help researchers” and “prevent postpartum depression.” While altruism may have temporarily inspired women to complete the screening surveys, this motivation perhaps waned when it was time to actually participate.

The original study design included the collection of biological fluids (blood and urine) to corroborate dietary intake and BPA exposure data. Other researchers have noted that pregnant women are reticent to provide biological specimens [56, 57]. The findings of this previous work are supported in the current study by the increased number of completed screening surveys once biospecimen collection was removed from the study protocol.

The fact that traditional recruitment methods yielded the highest number of participants is not surprising because previous research has found that personable, enthusiastic, non-judgmental, and empathetic recruitment personnel and research staff aid in recruitment efforts [6, 58]. Others have shown that recruitment was also improved by working with obstetricians for participant referrals [3, 12, 53, 54], as in the current study. Although Gatny and Axinn [54] suggest that health education may motivate women to participate, we were unable to successfully recruit potential participants by offering free early childhood nutrition and feeding classes. While it is time consuming to travel to businesses and medical offices, meet with office managers, and distribute study materials, this personal touch made a difference and, combined with word-of-mouth recruitment, proved worth the investment.

A personal connection may also explain our success using institutional broadcast emails. The primary benefit to the broadcast email approach is that it took little time compared to the traditional approach, yet yielded a similar participation rate. When considering the entire sample, women recruited via broadcast email were more likely to be eligible for the study. Importantly, women that completed the screening survey as a result of traditional recruitment methods or broadcast email were more likely to actually participate in the study. Other studies report employing broadcast emails but do not analyze this category separately, generally grouping email along with social media into “electronic” or “online” recruitment [59]. We chose to examine broadcast email as a separate category because the large number of individuals reached at our institution (approximately 4000) by a single email was greater than the paid reach of all but one of our paid Facebook posts.

While the literature is somewhat discordant regarding the use of social media for recruitment with studies showing greater, similar, and lower efficacy than traditional methods, a pattern emerges when the time commitment on the part of the participant is considered. We hypothesize that social media recruitment results in a large number of participants when studies require relatively little time to complete. For example, social media is an excellent recruitment tool when study participation requires very little of the participants’ time to complete a survey that can be reached with one click on a Facebook post [60, 61]. To wit, Admon et al. [61] recruited 5 times more individuals using Facebook than in-clinic recruitment to participate in a survey-based study requiring only 15 min to complete. Similarly, another study showed the enormous reach of social media by recruiting 1075 women in early pregnancy over an 18-week period to respond to an online survey, 10–15 min in duration, regarding childbirth preferences [60]. The screening survey for the FFF Study required only 10–15 min to complete and could be reached via one click from our Facebook posts. Reflective of this, social media was the most effective recruitment method, responsible for over half of the women that completed the screening survey, but a different story emerges with respect to participation. Participation in the FFF Study entailed at least 2 h of the participants’ time spread over multiple days to complete. Importantly, women recruited via social media were less likely to participate than women recruited via traditional methods or via broadcast email. While it is not possible to know why eligible women failed to participate in the study, we can speculate. For example, we offered an incentive of $100, for our subjects with relatively high income, this amount of money may not have been sufficient to secure commitment to the study. Alternatively, women may have lacked time.

High interest, followed by lower participation rates for potential participants found through social media, termed “conscientious recruits” by Frandsen [62], is emerging as a common problem when studies require a longer time commitment, for example randomized controlled trials examining prevention of weight gain [63] and smoking cessation drug efficacy [62]. Adam et al. [64] recruited almost 10 times the number of pregnant women per day using social media than through traditional recruitment methods similar to those employed in the current study, but also found that women recruited via social media were less likely to participate in their trial comparing counseling to standard care for management of appropriate pregnancy-related weight gain. Similarly, Christensen et al., [65] recruited women trying to conceive to a nutrition and lifestyle study. They used offline and online methods to recruit, including Facebook ads. While they conclude that online recruitment was more successful than offline, only 3.6% of their actual participants were recruited via Facebook advertisements. Finally, Van Gelder et al., [66] reported greater success with traditional methods than paid Facebook promotions regarding recruitment of pregnant women into their prospective, long-term longitudinal study. They also report, similar to the current study, lower participation rates among women recruited through Facebook vs traditional methods. In summary, recruitment via social media can be successful when only a short period of attention is required to complete the study, as with survey-based research. When considering the larger time commitments required of randomized controlled trials or longitudinal studies, recruitment through social media may be detrimental in terms of both time and financial investments.

To our surprise, most of the women recruited through social media were not a result of paid promotions. Rather, these women completed the screening survey because they saw our unpaid posts on groups they were members of. At a cost of almost $7 per completed screening survey, the cost effectiveness of paid social media promotions is questionable. In contrast to our results, Bennetts et al., [67] reported far greater success with paid posts than unpaid. Their success may be due to the fact that the first part of their study involved a short time commitment, so participants were likely to click the link and complete this survey portion of the study when using Facebook. Importantly, when contacted to complete a second, follow-up survey, Bennetts et al., [67] note that participation fell by almost half, supporting our hypothesis that social media is most useful for short, one-time only survey-based research.

Previous studies have reported that participants recruited through social media tend to be white [68–70], more educated [69, 71–73], and affluent [74] when compared to a reference population. Given the small sample size of the current study, a comparison to a reference population is not instructive. Within the current study, these parameters did not differ by recruitment method.

As mentioned above, women with perinatal depression are difficult to recruit to research studies (systematically reviewed in [14]); therefore, the EPDS scores of participants at the time of recruitment should be considered. Scores of 13 or greater on the EPDS have traditionally been thought to indicate risk for pregnancy-related depression [28]. More recently, in recognition of the fact that depression is not binary, the EPDS score has been used in a more continuous manner, with increasing scores corresponding to a greater likelihood of depression [75]. In this new system, EPDS scores of 0 to 6 are thought to reflect no or minimal depression while scores of 7 to 13 are indicative of mild depression. Collectively, our participants would be categorized as having minimal depression based on their responses to the EPDS survey administered during screening. Subsequent publications from our group will explore how EPDS scores change over time and relate to diet in this cohort.

While informative, this study had several limitations. Firstly, our group had no prior experience with paid Facebook promotions. Although we consulted with a social media expert, with additional experience, the posts could possibly have been improved to better target pregnant women, however this would have required market research which was beyond the scope of this pilot study. Secondly, the sample size was small for two primary reasons revolving around technology access and the time commitment required to complete the study. Specifically, among women recruited via social media and email, the most common reason for ineligibility was lack of access to a computer with a webcam and microphone. This barrier highlights the importance of adapting studies, when possible, to accommodate participation through a more ubiquitous device, such as a smartphone. For the present study, it was not possible to use a smartphone because our cognitive test platform, PEBL, required the use of a Windows or Macintosh operating system. Finally, although not a limitation, the relatively large amount of time participants must devote to longitudinal studies is a challenge to recruitment that should be considered.

## Conclusions

Social media has the potential to reach an enormous number of individuals, particularly in hard to recruit populations such as women experiencing pregnancy-related depression. Unfortunately, for women recruited through social media, follow through resulting in full participation in the FFF study was low. In short, recruitment though social media may not be the best recruitment method for studies involving a relatively large time commitment. Rather, a personal connection, for example between study personnel and healthcare professionals, as with traditional recruitment methods, or institutional loyalty, as seen following broadcast email, promotes participation particularly in studies requiring a large time commitment.

## Supplementary Information

**Additional file 1:.** January 2019 FFF Study Newsletter. sample newsletter sent to stakeholders and participants.

**Additional file 2:.** February 2019 FFF Study Newsletter. sample newsletter sent to stakeholders and participants.

**Additional file 3: Table S1.** Facebook groups and pages joined or contacted to promote FFF Study. list of Facebook groups.

## Data Availability

Data may be made available by the corresponding author upon a reasonable request.

## Abbreviations

FFF: Food, feelings, and family
BPA: Bisphenol A
EPDS: Edinburgh postpartum depression survey
PEBL: Psychology experiment building language
