# Supplementary material for: The Food, Feelings, and Family Study: comparison of the efficacy of traditional methods, social media, and broadcast email to recruit pregnant women to an observational, longitudinal nutrition study
Source: BMC Pregnancy Childbirth. 2021 Mar 12;21:203. doi: 10.1186/s12884-021-03680-1 (PMC7953646; doi:10.1186/s12884-021-03680-1)
Supplement: Supplementary file 1 — Additional file 1:. January 2019 FFF Study Newsletter. sample newsletter sent to stakeholders and participants. [file 12884_2021_3680_MOESM1_ESM.pdf]

JANUARY, 2019

OFFICIAL NEWSLETTER OF THE FOOD, FEELINGS, AND FAMILY RESEARCH TEAM

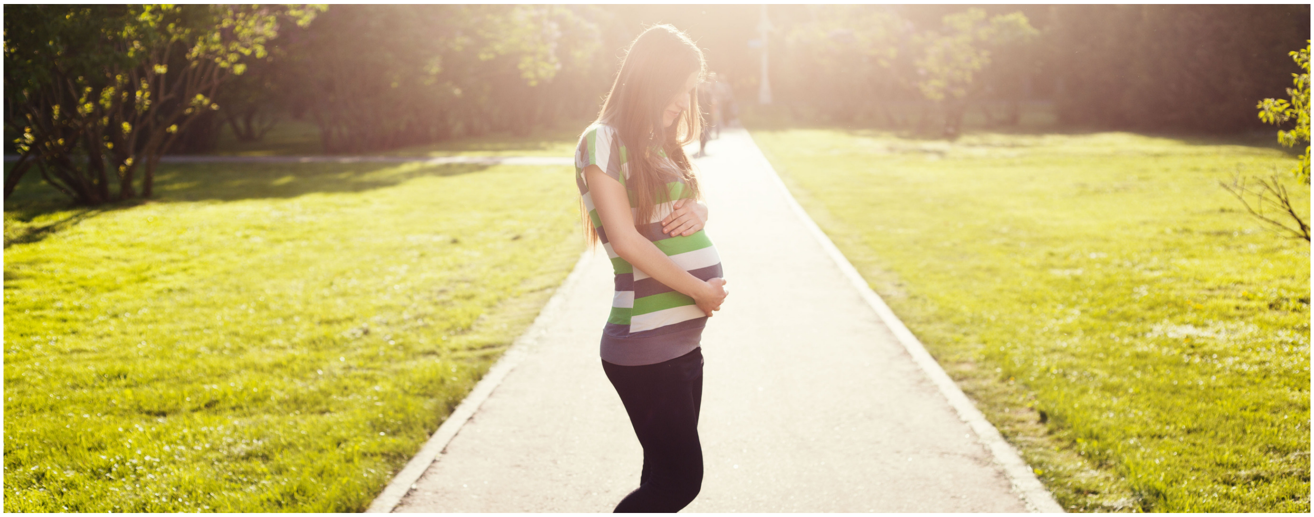

UPCOMING FREE CHILDHOOD NUTRITION CLASSES:

- Jan 24th 12:30 pm - 1:30 pm Kyle Library Room B 550 Scott St, Kyle, TX 78640
- Feb 2nd 11 am to 12 pm Mueller District, Red Cross Building B Classroom A 2218 Pershing Dr, Austin, TX 78723
- Feb 16th 11 am to 12 pm FCS building, Room 183, Texas State University, Academy St, San Marcos, TX 78666

INFORMATION OVERLOAD: WHAT SOURCES CAN I TRUST?

By Regina Gillman, Graduate Assistant

Do you feel like you are receiving information everywhere you look? Whether it is on Facebook, a television advertisement, or a celebrity testimonial claiming “This product is AMAZING and will help you with X, Y, Z...”. Women are given a lot of advice and information during pregnancy. It can sometimes feel overwhelming to figure out what information is reliable, or based on scientific evidence, and what is either nonsense or a big stretch. The Food, Feelings, and Family Research Team is here to help you identify reliable information that you can use during your pregnancy. Reliable information is factual, based on evidence, and provided by experts. Sometimes people who are not experts will attempt to appear as experts in order to make money.

How can we tell what is reliable information?

- Reliable information presents the facts on a topic, rather than opinions.
- Authors of reliable information should hold the appropriate credentials in a field related to the information. For example, Registered Dietitians/ Registered Dietitian Nutritionist (RD/RDN) are the top experts in nutrition.
- The American Academy of Pediatrics, The American College of Obstetricians and Gynecologists, The American Pregnancy Association, and other professional medical organizations are excellent sources of evidence-based, reliable information for pregnant women.
- The Food, Feelings, and Family Research Team consists of nutrition professionals, including individuals with Doctoral degrees and RD credentials.

What are signs of non-reliable information?

- Sources that rely on testimonials, anecdotes or offer 'miracle' cures and quick fixes are often not reliable and should be read with a skeptical view.
- Sources that promote particular products or are funded by a product's developers are also not reliable and typically have little evidence supporting them.

"A lack of energy during pregnancy is often triggered by the hormonal changes that happen during pregnancy. The primary hormone affecting this change in energy levels is progesterone." - http://americanpregnancy.org/pregnancy-health/lack-energy-pregnancy/
This is reliable information because it comes from a credible source, presents factual information rather than an opinion, and is not attempting to sell you anything.

"If you purchase Happy Morning, it will cure morning sickness! I used this during my pregnancy and it was amazing! Purchase Happy Morning by clicking this link." -Blog (not a real product)
This should not be considered reliable information because the statement is meant to persuade you into purchasing this product. Also, an author of a blog may not have the proper credentials.

Additional Resources

- https://www.acog.org/Patients
- http://americanpregnancy.org/pregnancy-health/
- https://www.fcs.txstate.edu/ms_nutrition/faculty/lane/ffftxst.html

Who are we?

Food, Feelings, and Family is a research team at Texas State University that is working to determine how what you eat during pregnancy affects the way you feel during and after pregnancy.

Interested in participating?

Visit our website for more information or text your email to (512) 670- 8405 to determine if you are eligible!

https://www.fcs.txstate.edu/ms_nutrition/faculty/lane/ffftxst.html

Food Feelings Family
