# Supplementary material for: The Food, Feelings, and Family Study: comparison of the efficacy of traditional methods, social media, and broadcast email to recruit pregnant women to an observational, longitudinal nutrition study
Source: BMC Pregnancy Childbirth. 2021 Mar 12;21:203. doi: 10.1186/s12884-021-03680-1 (PMC7953646; doi:10.1186/s12884-021-03680-1)
Supplement: Supplementary file 2 — Additional file 2:. February 2019 FFF Study Newsletter. sample newsletter sent to stakeholders and participants. [file 12884_2021_3680_MOESM2_ESM.pdf]

FEBRUARY, 2019

OFFICIAL NEWSLETTER OF THE FOOD, FEELINGS, AND FAMILY RESEARCH TEAM

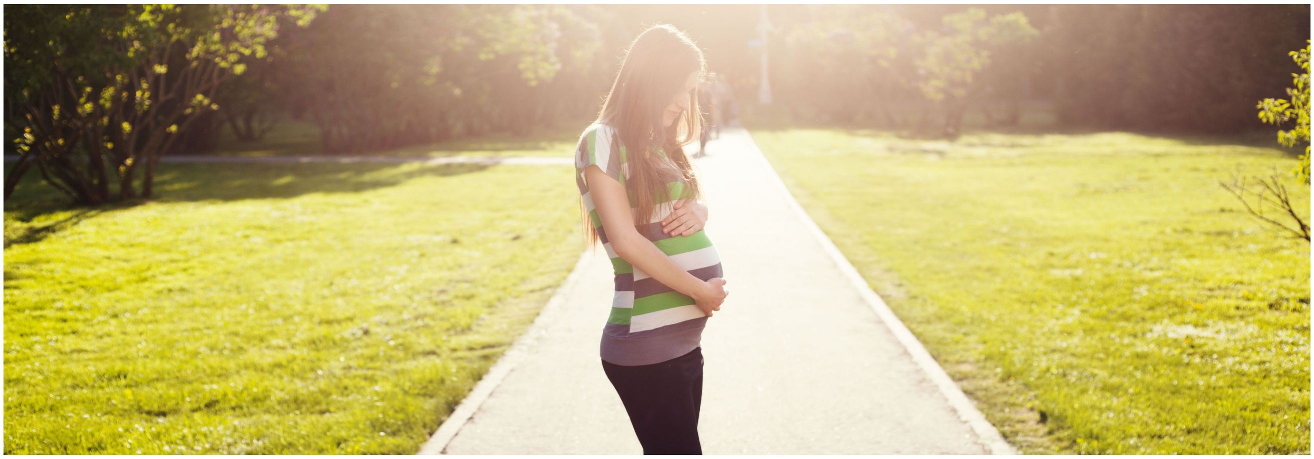

FIRST FOODS & IRON

By Regina Gillman, Graduate Assistant

Weaning, often referred to as complementary feeding, is the process of adding foods other than breastmilk or formula into an infant's diet. While breastfeeding should remain the primary source of nutrition for infants, breastmilk can no longer meet all of the infant's nutritional needs by the time he or she is around 6 months of age. Thus, it is appropriate to begin introducing solid foods at around this time. More specifically, no complementary foods should ever be offered before the age of 4 months, and it is important to get things started at around 6 months at the latest. Introducing solid foods too early (before 4 months) may put infants at risk for gaining excess weight and increase risk for choking. Introducing foods late (after 7 months) may increase risk for picky eating and developing deficiency of important nutrients.

To figure out when to begin offering complementary foods, parents and caregivers should look to the infant for cues.

Every infant is different! In general, a baby signals readiness to start eating by being able to sit upright, without support, showing an interest in foods, bringing food to the mouth, and keeping food in the mouth without spitting it out as a reflex. Learning to eat is a process and that takes time for infants to master. Be patient! Offering healthful foods in a supportive manner is incredibly important for the infant's growth, nutrition status, and even future social and emotional behaviors. The process of eating solid foods helps infants improve their coordination and teaches them to enjoy a variety of foods if they are offered. While it is typical to start feeding just a few foods, in time, the caregiver will want to add more. Keep in mind that an infant may need to be exposed to a new food up to 15-30 times before accepting it. Don't be discouraged! While it may be intimidating for caregivers when infants seem to reject certain foods, it is important to be persistent so that the infant is regularly exposed to many different tastes and textures. At some point, they may surprise you!

When they do start offering foods to babies, caregivers should choose foods that are nutritious and safe. Nutritious foods often include fruits and vegetables, all kinds of beans, and lean meats such as baby food meats, fish, shredded chicken, and baby food cereals. Safe foods include those that do not pose a chocking hazard. Foods that are not nutritious include processed foods like chips, sodas, desserts, candy, white bread, etc. Often caregivers begin using a spoon to offer pureed or slightly lumpy foods, and progress to finger foods as an infant is able to pick them up. Caregivers should always avoid foods that can cause choking, such as cut up hot dogs, nuts, grapes, and other large pieces of food that may get stuck in the throat.

During the first few months of weaning, the most important nutrient of concern is iron. This is because infants usually have enough iron stores to last them until they are 4-6 months, and after that time, breast milk does not provide enough iron. This is an emergency! Because breastmilk is naturally low in iron, caregivers should make sure that they offer many complementary foods that are rich in iron. Iron-rich foods include eggs, pureed baby food meats, fish, beans, lentils, and fortified infant cereals. When choosing iron-rich foods, it is important to also include foods that are high in vitamin C, such as citrus fruits, which increase iron's ability to be absorbed by the infant's body . Foods high in vitamin C, such as many fruits and vegetables, increase the absorption of iron. Infants who do not consume enough iron are at risk for deficiency, which may impair brain function.

Do you want more resources on how to introduce solid foods to infants? While there are currently no official guidelines in the United States, the Centers for Disease Control (CDC) and the Robert Wood Johnson Foundation are excellent resources! Find them underneath our additional resources!

Additional Resources

- https://www.cdc.gov/nutrition/infantandtoddlernutrition/index.html
- https://www.rwjf.org/

Who are we?

Food, Feelings, and Family is a research team at Texas State University that is working to determine how what you eat during pregnancy affects the way you feel during and after pregnancy.

Interested in participating?

Visit our website for more information or text your email to (512) 670- 8405 to determine if you are eligible!

https://www.fcs.txstate.edu/ms_nutrition/faculty/lane/ffftxst.html
