# Supplementary material for: The Food, Feelings, and Family Study: comparison of the efficacy of traditional methods, social media, and broadcast email to recruit pregnant women to an observational, longitudinal nutrition study
Source: BMC Pregnancy Childbirth. 2021 Mar 12;21:203. doi: 10.1186/s12884-021-03680-1 (PMC7953646; doi:10.1186/s12884-021-03680-1)
Supplement: Supplementary file 3 — Additional file 3: Table S1. Facebook groups and pages joined or contacted to promote FFF Study. list of Facebook groups. [file 12884_2021_3680_MOESM3_ESM.docx]

| **Supplemental Table.** Facebook groups and pages joined or contacted to promote FFF Study. | |
| --- | --- |
| Facebook Groups or Pages by Category |  |
| *Community Groups* |  |
| Early Childhood Coalition Hays County |  |
| Splendora Folks |  |
| Plum Creek- Kyle |  |
| *Health Care Providers* |  |
| Austin Area Birthing Center |  |
| Birth Center Stone Oak |  |
| Beautiful Beginning Birth Center |  |
| Central Texas Birth Center |  |
| Austin Doula Care |  |
| Doulas of Austin |  |
| Austin Midwife Collection |  |
| Austin Doula Collective |  |
| Austin Midwife 2 U |  |
| Networx Health |  |
|  |  |
| *General Mental Health Groups* |  |
| Depression and Anxiety Talk |  |
| Depression and Anxiety |  |
| Depression and Anxiety Mental Health Support |  |
| Depression/Mental Health Group |  |
| Mental Illness |  |
|  |  |
| *Perinatal depression* |  |
| Perinatal Mood Disorder |  |
| Mothers/Women with Depression and Anxiety |  |
|  |  |
| *Pregnancy/Motherhood* |  |
| Mommy Support |  |
| Pregnant Mom's Due in 2019-2020 |  |
| Moms of Kingwood |  |
| Moms and Moms to Be |  |
| Bellies, Babies, and Mommies |  |
| Moms of Houston |  |
| "Real Moms" of New Braunfels |  |
| Kyle, Buda, San Marcos Moms |  |
| Mommy Rules |  |
| Mommy's Page |  |
| Mothering San Marcos |  |
